# Red deer (*Cervus elaphus*) Did Not Play the Role of Maintenance Host for Bluetongue Virus in France: The Burden of Proof by Long-Term Wildlife Monitoring and *Culicoides* Snapshots

**DOI:** 10.3390/v11100903

**Published:** 2019-09-27

**Authors:** Sophie Rossi, Thomas Balenghien, Cyril Viarouge, Eva Faure, Gina Zanella, Corinne Sailleau, Bruno Mathieu, Jean-Claude Delécolle, Camille Ninio, Claire Garros, Laëtitia Gardès, Christophe Tholoniat, Agnès Ariston, Dominique Gauthier, Stevan Mondoloni, Aurélie Barboiron, Maryline Pellerin, Philippe Gibert, Corinne Novella, Stéphane Barbier, Etienne Guillaumat, Stéphan Zientara, Damien Vitour, Emmanuel Bréard

**Affiliations:** 1 ONCFS, research department, wildlife diseases unit, 78610 Le Perray-en-Yvelines, France, sophie.rossi@oncfs.gouv.fr (S.R.); philippe.gibert@oncfs.gouv.fr (P.G.); elo-stef-barbier@orange.fr (S.B.); 2 CIRAD, UMR ASTRE, F-34398 Montpellier, France, claire.garros@cirad.fr (C.G.); laetitia.gardes@cirad.fr (L.G.); 3 ASTRE, Univ Montpellier, CIRAD, INRA, F-34398 Montpellier, France; 4 IAV Hassan II, UR MIMC, 10100 Rabat, Morocco; 5 UMR Virologie, INRA, Ecole Nationale Vétérinaire d’Alfort, laboratoire de santé animale d’Alfort, ANSES, Université Paris-Est, 94700 Maisons-Alfort, France, cyril.viarouge@anses.fr (C.V.); gina.zanella@anses.fr (G.Z.); corinne.sailleau@anses.fr (C.S.); stephan.zientara@anses.fr (S.Z.); damien.vitour@anses.fr (D.V.); 6 National hunters Federation, 92130 Issy-les-Moulineaux, France; efaure@chasseurdefrance.com; 7 IPPTS, Université de Strasbourg, EA 4438, 67000 Strasbourg, France; bmathieu@unistra.fr (B.M.); jean-claude.delecolle@numericable.fr (J.-C.D.); 8 USC Vecpar, ANSES-LSA, Université de Reims Champagne-Ardenne, SFR Cap Santé, Faculté de Pharmacie, 51 rue Cognacq-Jay, EA 4688, 51096 Reims, France, camille.ninio@gmail.com; 9 Regional farmers sanitary support, Region Centre, 36000 Chateauroux, France; ctholoniat@gdscentre.fr; 10 Veterinary diagnosis laboratory, 05000 Gap, France; agnes.ariston@hautes-alpes.fr (A.A.); dominique.gauthier@hautes-alpes.fr (D.G.); 11 Regional natural park of Corsica, 20250 Corte, France; smondoloni@pnr-corse.fr; 12 ONCFS, research department, wild ungulates unit, 55000 Bar-le-Duc, France; aurelie.barboiron@oncfs.gouv.fr (A.B.); maryline.pellerin@oncfs.gouv.fr (M.P.);; 13 Veterinary diagnosis laboratory, 64150 Lagore, France; c.novella@labopl.com; 14 Veterinary practice «clinique vétérinaire des trois sources», 21320 Pouilly-en-Auxois, France; 15 National Domain of Chambord, Forest direction, 41250 Chambord, France; etienne.guillaumat@chambord.org

**Keywords:** arboviruses, vector-borne disease, wildlife, neutralizing antibodies, Europe

## Abstract

Bluetongue virus (BTV) is a *Culicoides*-borne pathogen infecting both domestic and wild ruminants. In Europe, the Red Deer (*Cervus elaphus*) (RD) is considered a potential BTV reservoir, but persistent sylvatic cycle has not yet been demonstrated. In this paper, we explored the dynamics of BTV1 and BTV8 serotypes in the RD in France, and the potential role of that species in the re-emergence of BTV8 in livestock by 2015 (i.e., 5 years after the former last domestic cases). We performed 8 years of longitudinal monitoring (2008–2015) among 15 RD populations and 3065 individuals. We compared *Culicoides* communities and feeding habits within domestic and wild animal environments (51,380 samples). *Culicoides* diversity (>30 species) varied between them, but bridge-species able to feed on both wild and domestic hosts were abundant in both situations. Despite the presence of competent vectors in natural environments, BTV1 and BTV8 strains never spread in RD along the green corridors out of the domestic outbreak range. Decreasing antibody trends with no PCR results two years after the last domestic outbreak suggests that seropositive young RD were not recently infected but carried maternal antibodies. We conclude that RD did not play a role in spreading or maintaining BTV in France.

## 1. Introduction

Bluetongue virus (BTV) is a *Culicoides*-borne pathogen that may infect several ruminant species, including both domestic and wild animals [1,2]. This raises the question of the possible maintenance of BTV in sylvatic cycle, which is well known in Northern American wildlife but not yet demonstrated in Europe [3,4]. The maintenance of sylvatic cycle requires: (i) Wild ruminants as amplificatory hosts, i.e., able to develop a sufficiently long and high viremia to infect *Culicoides* females; (ii) competent *Culicoides* vector species able to bite them; and (iii) sufficient interactions between the infected wild ruminants and sylvatic-associated *Culicoides* vector species to ensure epidemiological cycles in natural environments.

The role of *Culicoides* as vector species in wildlife has been limitedly investigated in the Palearctic region, excepted in relation to avian parasites transmission in wild bird populations [5]. In Europe, entomological studies have been carried out in both farms and game preserves in Spain [6] and in the Czech Republic [7]. In Spain, the species composition seems more influenced by the climate (dominance of *Culicoides imicola* under the southern Mediterranean climate, and of morphologically close species *Culicoides obsoletus s.l.*/*Culicoides scoticus* under the northern oceanic climate) than by the host species or by the farm/game preserve environment [6]. In the Czech Republic, species belonging to the *Avaritia* and, at a much lesser extent, to the *Culicoides* subgenera were dominant in farms (~97% of the collected *Culicoides*) and abundant in game preserves (~64%). Game preserves were associated with forest species such as *Culicoides pallidicornis* (~19%), *Culicoides furcillatus* (~3%), and *Culicoides festivipennis* (2%), often rare in farms [7]. These studies demonstrated that, under Mediterranean, oceanic, or wet continental climates, several *Culicoides* species, recognized as probable BTV vectors and belonging to the *Avaritia* and *Culicoides* subgenera, could be abundant in both farms and game preserves [6,7], where they are able to feed on wild ruminants [8]. These results allowed hypothesizing that *Culicoides* associated with domestic ruminants and farm environments are able to act as bridge vectors and transfer BTV from domestic to wild ruminant populations. However, little is known about *Culicoides* species associated with natural environments, which may be able to maintain the virus in sylvatic cycles involving only wild ruminants.

At the European level, the Red Deer (*Cervus elaphus*) (RD) has been considered a potential BTV “reservoir” [9]. Indeed, RD populations have been widely exposed to different BTV strains all over Europe since the 2000s, as demonstrated by serological surveys [10,11,12,13]. Natural populations exhibited positive PCR results during wintertime in areas where livestock had been exhaustively vaccinated [13]. They exhibited relatively long positive PCR results when experimentally infected with BTV serotype 8 (BTV8) or BTV1 [14]. 

**Figure 1:**
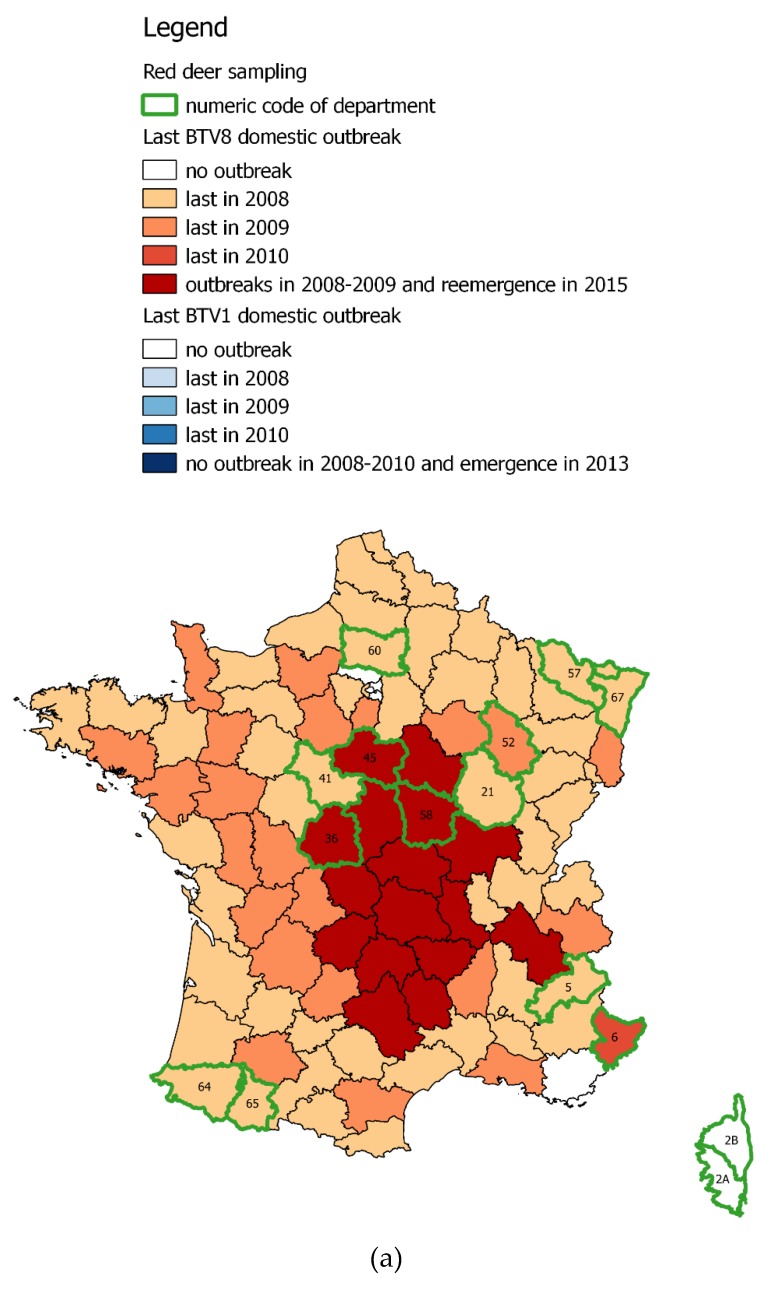

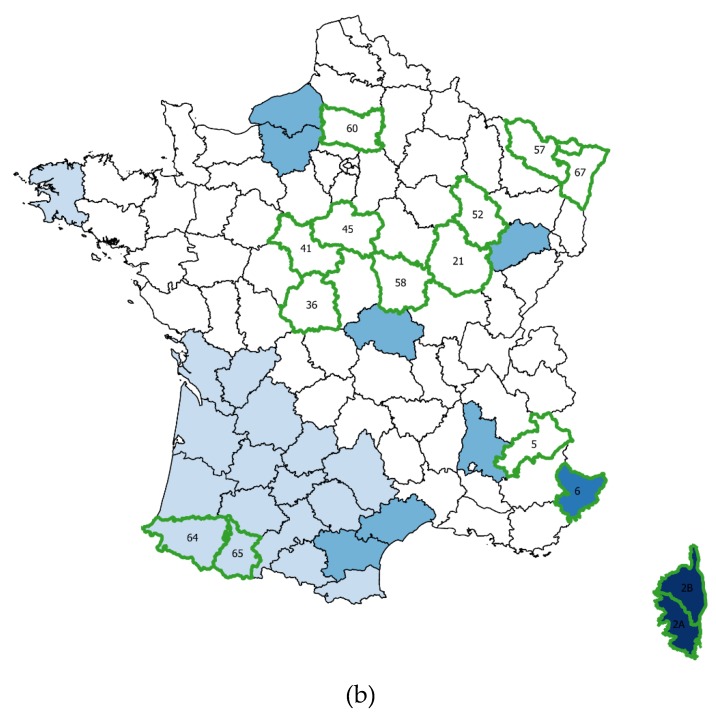
Localization of occurrence of outbreaks in domestic livestock from 2008 up to 2015. (**a**) BTV8; (**b**) BTV1.

These two last serotypes have been the cause of many serious animal epidemics in Europe, particularly in France. In 2006, BTV8 was reported in Europe for the first time, detected at the border between the Netherlands, Belgium, and Germany. BTV8 quickly spread to Western and Central Europe between 2006 and 2008 [15]. The disease progressed over the 2007/09 period in mainland France according to an epizootic wave from north–east to south–west, resulting in more than 43,000 infected holdings in all departments in mainland France (Figure 1) [16]. Simultaneously, BTV1 was introduced in 2007, but remained mainly limited to the south-west of the country in 2007/08 (and some cases in French Brittany), infecting about 4500 holdings (Figure 1) [15]. Inactivated BTV vaccines became available in 2008, allowing two compulsory vaccination campaigns in 2008/09 and 2009/10 against both serotypes in mainland France. Mainland France regained its BTV-free status by 2012. In August 2015, a BTV8 case was reported in Central France [17]. Phylogenetic studies showed that BTV8 genome sequences were closely related to the BTV8 strain that circulated in Europe in 2006–2008 [18]. It is likely that the origin of this re-emergence is due to a very low-level circulation of BTV8 either in domestic, wild ruminant, or both since its first emergence in 2006 [19]. The situation observed on the Mediterranean Corsica Island was different from the continent. From 2000 to 2003, three BTV serotypes emerged in Corsica: BTV2, BTV4, and BTV16 [20]. From 2004 to 2013, no domestic outbreaks occurred, but by September 2013, BTV1 emerged on the island, causing 169 outbreaks [20,21]. Since a compulsory vaccination campaign against BTV1 in 2013–14, no more BTV1 cases have been reported in Corsica (Bréard Com. Pers.).

Studies exploring BTV seroprevalence in wild cervids in Southern and Central Spain have reported the persistence of BTV in RD for more than 5 years in some areas, and proposed the hypothesis of the maintenance role of this species in the Iberian Peninsula [9,22,23,24]. The *Culicoides* fauna of these Mediterranean areas is known to be dominated by *C. imicola*, a proven BTV vector [6]. In continental France, outside the distribution range of *C. imicola* (only present with high abundance populations on Corsica Island), antibodies have also been described in young RD several years after livestock vaccination, suggesting a potential BTV maintenance in this non-vaccinated species [13,25]. The unexpected re-emergence of BTV8 reported in France in August 2015 in domestic livestock raised again the question of the role of RD in BTV persistence in the continental areas. 

In the present study, we thus explored the epidemiological role played by the RD (1) in BTV spreading out of the domestic outbreak range, (2) in maintaining a long-term sylvatic cycle in both Continental France and Corsica, and (3) in being a source of virus explaining the recent BTV8 re-emergence observed in livestock in continental France.

We first described the evolution of raw seroprevalence (ELISA results) and virological evidences (PCR results) regarding BTV8 and BTV1, in order to discuss the match between wild and domestic outbreaks and the spatiotemporal trends of prevalence in RD. Given the few positive PCR data during the past years, we focused our analyses on seroprevalence trends and on neutralizing antibody (NA) titers. In addition, we performed, in different non-Mediterranean eco-climatic zones, a comparative characterization of *Culicoides* species communities occurring in natural areas (with few anthropization processes) used by RD and other wild ruminant species (mostly forest environments) with those occurring in farms or pastures close to livestock.

## 2. Material and Methods

### 2.1. Wildlife Investigations

#### 2.1.1. Wildlife Sampling

**Figure 2:**
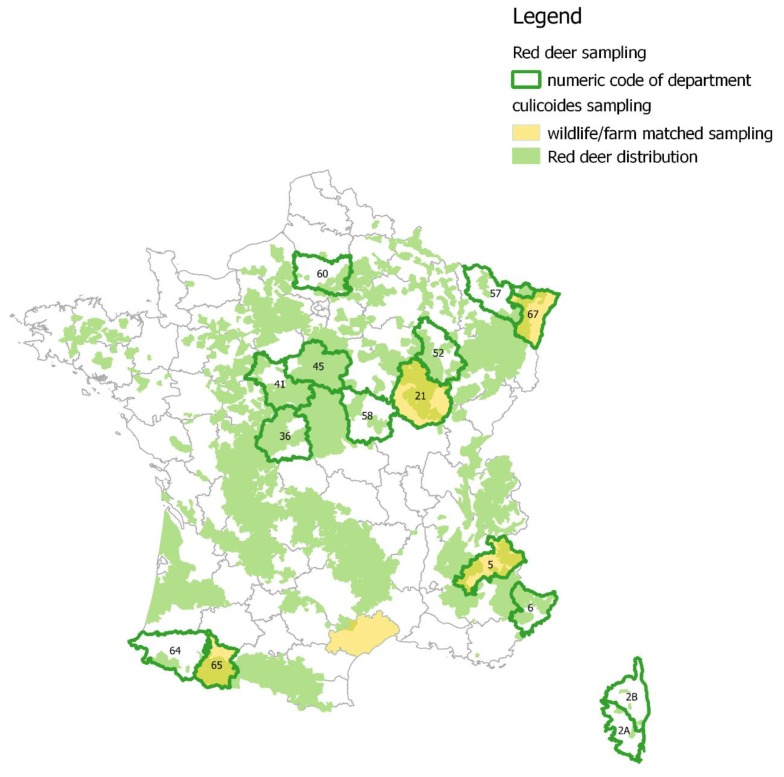
Localization of red deer and *Culicoides* samples.

We used RD sera, spleen, and full blood samples collected during previous studies implemented in France from 2008 to 2015 (Table 2, Figure 2). Samples were collected from 2008 to 2015 according to (i) long-term BTV monitoring performed by the French National Hunting and Wildlife Agency (ONCFS) [13,25], (ii) sera/organ banks constituted by the hunter and farmers federations, and (iii) research programs realized in the Natural Park of Corsica, the Chambord National Domain. RD is a hunted species in continental France, whereas in Corsica, the local subspecies (*Cervus elpahus corsicanus*) has been recently restored and is thus not yet hunted [26]. Local authorities authorized captures in three estates in the Corsica region, in la Petite Pierre (Alsace region), and in Chambord (Centre region), according to national and European legislation, either using mechanical or chemical contention [27]. Most of these sera were stored at −80 °C [13,25]. Among the sera stored at −20 °C provided by the hunter federations, we used only the samples collected from 2013 to 2015 in order to limit the risk of immunoglobulin degradation over time [28].

#### 2.1.2. Diagnostic Tests for BTV

Different laboratories were involved in the BTV monitoring: The public veterinary laboratories localized in each department (LVD) and the National Reference Laboratory (NRL) (National Food Safety Agency, ANSES, Maisons-Alfort). Serological analyses were made on each serum sample using competition ELISA (c-ELISA) commercial kits. These c-ELISA kits were used as per the manufacturer’s instructions for the detection of BTV VP7 antibodies in all serum samples. Virus genome detection was only implemented for animals exhibiting a positive or doubtful serological result by performing real-time RT-PCR (RT-qPCR) on spleens (hunted animals) or on blood (captured animals) using commercial kits, detecting all serotypes (Thermofisher^®^ or Biox^®^). Briefly, total RNA from spleen or EDTA-blood samples (using ethylenediaminetetraacetic acid (EDTA) as anticoagulant) were extracted using a MagVet™ Universal Isolation kit (Thermo Fisher Scientific, Lissieu, France). Total RNA was eluted into 80μL; 5 µL of denatured RNA was then tested using the RT-qPCR method. Commercial RT-qPCR kits were used for pan BTV detection (Adiavet^TM^ BTV kit (Bio-X Diagnostics, Saint Brieuc, France)) and for the detection of BTV1 and 8 (VetMAX™ European BTV Typing Kit), as per the manufacturer’s instructions. In the c-ELISA positive animals, sera were tested using a virus neutralization test (VNT), as described previously [29], targeting BTV1, BTV8, or both, within the known historical range of each serotype in domestic and wild animals. Both serotypes were also tested for a panel of sera from a department where BTV1 or BTV8 had not been recorded (e.g., departments 21, 36, 41, and 52 were tested regarding the BTV1 strain while having no recorded domestic BTV1 outbreak) in order to compare the spatial distribution of both BTV strains among wild and domestic populations. 

#### 2.1.3. Risk Factors

We considered the risk factors of BTV exposure in RD at two levels: The individual and the population. At the individual level, age and gender were determined by hunters or scientific teams according to animal body mass, teeth eruption, and presence of genital organs. Three age classes were considered: Less than 12 months (young), between 12 and 24 months (subadults), and more than 24 months (adult). We defined year from April N to March N+1 for accounting for the different hunting and sampling techniques and being representative of a single vector activity season.

Each RD population was defined at the scale of the department, a French administrative unit from 2000 up to 10,000 km^2^. For each department, we recorded the occurrence of domestic outbreaks, either involving BTV1, BTV8, or both strains from 2008 to 2015 (Table 1, Figure 1). We also accounted for the presence of BTV8 in the domestic livestock by 2015 (i.e., at the early time of its re-emergence) to detect a potential difference in the prevalence or antibody dynamics in RD inhabiting these recently re-infected departments (Figure 2). Regarding this particular question, we defined two zones concerning continental departments (excluding Corsica Island where BTV8 never occurred) (Table 1, Figure 1):

1. Zone A: Departments located near the BTV8 re-emerging outbreak (i.e., departments with domestic cases in 2015), and where RD experienced an initial exposure to BTV8 [13] (Table 1, Figure 1).
2. Zone B: Departments located out of the vicinity of the re-emerging outbreak, and where RD experienced an initial exposure to BTV8 [13] (Table 1, Figure 1).

#### 2.1.4. Statistical Analyses of the Red Deer Serological Data

Statistical analyses were performed on the data collected among 12 continental departments where BTV8 genome had been observed in both RD and livestock in 2008 and 2009 [13]. Three departments experienced livestock BTV8 outbreaks in 2015 (Zone A) (Table 1, Figure 1). 

We considered two different serological variables:

- The individual serological status based on the c-ELISA result; and
- Among the seropositive samples, the mean VNT titer regarding the BTV8 strain.

Seroprevalence was studied using logistic mixed models; positive and doubtful c-ELISA results being encoded as 1 and negative results as 0. Antibody titers were studied using linear mixed models; the inverse of titer was considered as a proxy of the sera’s NA concentration and log-transformed for maximizing model fit to data (log(1/titer +1)). The effect of age, gender, year, and zone was tested. The year effect was tested as a continuous fixed variable (from one in 2008–2009 to eight for 2014–2015). The department effect was considered as a random effect (repeated sampling over years), accounting for the initial exposure to BTV [13]. 

Model selection was based on the Akaike Information Criterion, corrected for overdispersion and small samples size (QAICc). Starting from a “complete” model, including all explicative variables and the interaction between the year and age, we explored simpler models and retained the set of models for which the difference in QAICc (delta-QAICc) was less than 2 [30]. These analyses were performed using the R software [31] and the package MuMin [32].

### 2.2. Entomological Analyses

#### 2.2.1. Entomological Collections and Identification

*Culicoides* were collected in different ecological and climatic zones (Figure 2): (1) In la Petite-Pierre, a temperate broadleaf and mixed forest dominated by continental climate (Alsace region); (2) in Pouilly, a bocage landscape, i.e., mixed woodland and pasture, dominated by continental climate (Bourgogne region); (3) in Chaudun and Ristolas, a submountainous to subalpine zone, dominated by dry climate (Alps region); (4) in Caroux, an altitude Mediterranean forest, under Atlantic climatic influence (Haut-Languedoc region); and (5) in the Bazès preserve, a subalpine zone, dominated by wet climate (Pyrenees region).

In each zone, 1 to 10 collection sites (average of 7 sites per zone) were sampled using nightly operated UV-light/suction traps (Onderstepoort Veterinary Institute model). Collection sites were selected in each zone along an environmental gradient (altitude or forest cover closure). Collections were organized during the period of known *Culicoides* peak of abundance, i.e., from late June to late August 2008, depending on the climatic zone. As much as possible, replicate collections were made the following nights (up to four).

The following years (from 2009 to 2012), UV-light/suction traps were run routinely across the French territory within the framework of the national *Culicoides* surveillance network [33]. They were all located in cattle or small ruminant farms. We selected one surveillance trap (located as close as possible to the collections carried out in the natural areas) in each of the ecological zones described above to compare *Culicoides* species communities in natural areas and in farm environments.

*Culicoides* species were identified morphologically down to species level using a reference manual and a morphological key [34,35]. *Culicoides lupicaris* was considered a valid species, despite the *Culicoides* world catalogue [36]. When blood-fed females were collected, the blood-meal origin was determined using the method described by Garros et al. [37]. Moreover, blood-fed females belonging to the morphologically close *Culicoides obsoletus* and *Culicoides scoticus* species were identified using molecular assay as described in [38].

#### 2.2.2. Description of *Culicoides* Species Communities in the Different Environments

As sampling design was not the same between sites, entomological data were aggregated against time. For each site, we first considered the highest number of *Culicoides* collected per species and per year, and then, if the site was sampled in several years, calculated the average of these yearly maxima. The *Culicoides* species communities were first described plotting graphs of log abundance on rank, the Renyi diversity profiles (renyi function), and the species accumulation curves (specaccum function on non-aggregated data, with the exact method) in each eco-climatic zone and for each trapping location (in natural areas or in farms). Observed and extrapolated (specpool function using the Chao equation) species richness and the alpha of log series (fisherfit function) were calculated, together with the Berger–Parker dominance and the evenness indices (diversity function). The global similarity between collection sites was assessed using a hierarchical cluster analysis (HCA, hclust function), using the Ward minimum variance method, on the Euclidian distance matrix of the log abundance (dist function). Then, we explored the structure of *Culicoides* species communities and relation between species in the collection sites using a principal component analysis (PCA, dudi.pca function) performed on the proportion of *Culicoides* per species and site transformed as ordinal classes (encoded 0 as absent, 1 as very rare (0–2%), 2 as rare (2–5%), 3 as secondary (5–20%), 4 as co-dominant (20–60%), and 5 as dominant (>60%)). For this latter step, we considered only sites for which at least 25 individuals were collected, and species for which the total abundance was superior to 100 individuals.

Ecological characteristics of the collection sites (the environment directly surrounding the trap location) were described according to the survey location as categorical variables (in natural areas or in farms), to the eco-climatic zone as categorical variables (WCF as wet continental forest, WCB as wet continental bocage, DHM dry high mountain, and WMM as wet medium mountain) and to the altitude as ordinal variable (<300 m as low, 300–500 m as medium, 750–1150 m as high, and >1700 m as very high).

The presence of cattle herd, small ruminant herd, or horses in the trap surrounding environment was noticed (encoded as 0 for absence and 1 for presence in three categorical variables). Moreover, we reported if the collection site was known to be frequented by RD, roe deer, or chamois (encoded as 0 for absence and 1 for presence in three categorical variables). Finally, the degree of contact between domestic and wild ruminants was estimated (encoded as null, rare, occasional, and frequent in a categorical variable); for instance, null if domestic animals were kept in a closed building or frequent in the case of cattle grazing in a bocage environment where roe deer are abundant. To avoid collinearity between these categorical variables and to limit the number of dimensions, we carried out a multiple correspondence analysis (ACM, dudi.acm function). Then, to obtain a limited number of comprehensive classes characterizing the host environment of the collection site, a HCA was carried out as described above on the ACM coordinates. Test values (vtest function) were calculated to describe the importance of each host variable to the partition. The test values are measurements of the distance between the within-class value and the overall value [39]. For continuous variables, test values compare the mean of *X* in the group *k* and the mean of *X* in the whole population, whereas for qualitative variable, test values compare the proportion of the population with the category *j* in a group *k* with the proportion of the population with the category *j* in the whole population.

The habitat in which collections occurred was described regarding the presence of crops or farm buildings in the close vicinity of the trap (encoded as 0 for absence and 1 for presence in two categorical variables). The degree of the landscape enclosure was assessed and encoded as 0 for closed, 1 for edge, 2 for mixed, and 3 for open in a categorical variable. Finally, the forest cover was characterized by the dominant tree species (encoded as absence for a completely open landscape without forest, larch, hardwood for mixed deciduous trees, beech, and conifers for mixed needle leaf trees in a categorical variable), and the herbaceous vegetation was characterized by the main vegetal association (encoded as absence for a completely enclosed landscape dominated by forest, dry meadow, humid meadow, moor, and peat bog in a categorical variable). Similarly to the host environment, we performed, as described above, an ACM on the habitat variables, then a HCA on the ACM coordinates to obtain a limited number of comprehensive habitat classes, and finally test values to describe the importance of each habitat variable to the partition.

The impact of the survey location, eco-climatic zone, altitude, host, and habitat classes on the global *Culicoides* species community was assessed using a between-class analysis (bca function) on the species community PCA. Basically, between-class analysis is a particular case of PCA, where only variability between groups is optimized [40]. This allows to estimate the impact of groups on the whole PCA, assessing the proportion of PCA inertia that is explained by the group partition. Then, the composition of *Culicoides* species in each class of these ecological drivers was described using test values on the log-transformed *Culicoides* abundances. Finally, a global overview of the results was given using a heat map (Heatmap function) carried out on the test values.

These analyses were performed using the R software [31] and additional packages: vegan [41], ade4 [42], tdisplay which includes the vtest function [43], and ComplexHeatmap [44].

## 3. Results

### 3.1. Red Deer Study

#### 3.1.1. Samples and Average Seroprevalence

Along the considered period (2008–2015), we aggregated data from 3065 RD with a conclusive c-ELISA result, from which 555 had a positive result (Table 2a). Among these 555 seropositive RD, 375 sera were correctly stored and could be examined and could deliver a conclusive VNT result as well: 331 regarding BTV8 and 103 regarding BTV1 (Table 3a,b). Fifty-two c-ELISA positive samples were selected in the banks from four continental departments with no confirmed BTV1 outbreaks in livestock and were tested regarding both BTV8 and BTV1 strains (detailed with an asterisk in Table 3b), and two c-ELISA positive samples were tested from Corsica with no confirmed BTV8 outbreaks in livestock (detailed with an asterisk in Table 3a). Among the 555 seropositive RD, 458 RT-qPCR examinations were performed, for which 176 were positive (positive samples are detailed in bold in Table 2b). It is interesting to note that 90.4% of the deer tested were positive for BTV8 in 2008–2009, and 30.4% in 2009–2010. Thereafter, all PCR tests were BTV8 negative.

Positive c-ELISA results were observed during the whole study period, and positive results in juvenile or subadult RD (i.e., less than 2 years old) were detected from 0 to 3 years after the last domestic case report, depending on the department (Table 2a). Seroprevalence gradually decreased from 2008 to 2015 in continental France, but increased in Corsica after 2013, i.e., after the emergence of BTV1 in livestock. 

Positive PCR results were observed in continental France from 2008 to 2009, and in Corsica in 2013, i.e., during the periods with concurrent domestic outbreaks (indicated in grey in Table 2) or the later year, depending on the department (Table 2b). The BTV serotypes identified in both RD and livestock were similar in the same departments and during the same periods (Table 1 and Table 2b). 

Serotype-specific antibodies against BTV1 or BTV8 were measured in a subsample comprising the departments where both strains had been detected in livestock, but also within a subsample of departments where only BTV8 had been confirmed in livestock (Table 3a,b). NA targeting BTV1 were not observed out of the known livestock infected areas in continental France, and were only observed after 2013 in Corsica. BTV8 and BTV1 NA were detected in one department in south-western France (department 64), where both serotypes had been reported in the domestic livestock as well (Table 1 and Table 3).

#### 3.1.2. Evolution of Seroprevalence (c-ELISA) and BTV8 NA Titers in the Continental France

Seroprevalence was explored using c-ELISA results from 2184 RD sampled in continental France with no missing data regarding risk factors. The best model retained the effect of the RD’s age, year, and interaction between the year, and age but not the effect of the zone (i.e., zone with or without BTV8 re-emergence) nor of the RD’s gender. Seroprevalence decreased over time, according to the same trend whatever the zone (OR_N+1/N_ = 0.377; CI (0.331; 0.429)). The initial exposure of fawns was lower than observed in subadult or adult RD (OR_juvenile/older_ = 0.250, 95%; CI (0.158; 0.396)). Seroprevalence decreased twice as quickly among young and subadult RD compared to adults (OR_juvenile&subadult/adults*year_ = 0.547, 95%; CI (0.477; 0.627)). Furthermore, no seropositive result was observed in animals less than two years of age after 2012 (Table 2).

NA titers against BTV8 were analyzed among 278 c-ELISA positive animals sampled in continental France with no missing data regarding the risk factors (Table 3c). The best model retained the effect of the year, and of the RD’s age and gender (Appendix A, Table A1 and Table A2). Antibody titer decreased over time, according to the same trend whatever the zone (delta-logVNT_N+1/N_ = −0.247; CI (−0.181; −0.314)) (Table 3c). Furthermore, titers were lower in males compared to females (delta-logVNT_male/female_ = −0.265; CI (−0.032; −0.498)) and lower in juvenile or subadult RD (less than 2 years old) compared to adults (>2 years old) (delta-logVNT_subadult/adult_ = −0.552; CI (−0.162; −0.942)).

### 3.2. *Culicoides* Species Communities

#### 3.2.1. *Culicoides* Collections

Among the five explored natural areas, 55 collections (from 1 to 26 per zone) were carried out in 34 different sites (from 1 to 10 per zone) from June to August 2008 (Table 4). A total of 51,380 *Culicoides* belonging to at least 30 species were collected. The most abundant species were the morphologically closely related species *C. obsoletus s.l./C. scoticus* (59.1% of the collected individuals), *Culicoides furcillatus* (17.7%), *Culicoides achrayi* (7.8%), *Culicoides punctatus* (6.1%), *Culicoides pallidicornis* (3.2%), *Culicoides kibunensis* (1.5%), and *Culicoides festivipennis* (1.5%). Altogether, these species represented 96.9% of the collected individuals. In the five selected farms, the most abundant species were *C. obsoletus s.l./C. scoticus* (76.3%), *Culicoides chiopterus* (4.9%), *C. achrayi* (2.9%), *Culicoides pulicaris* (2.6%), *Culicoides lupicaris* (1.9%), *Culicoides dewulfi* (1.4%), and *Culicoides vexans* (1.4%). The numbers of collected or expected species and the Fisher α on log-series were always higher in farms than in natural areas, but the sampling effort was much higher (Table 5). The diversity profiles were quite similar between sites [45], but diversity in farms was characterized by a large dominance of the *C. obsoletus s.l./C. scoticus* species, whereas in natural areas secondary species, such as *C. furcillatus*, *C. achrayi,* or *C. punctatus*, were found in significant numbers (Table 4). This was illustrated by Berger–Parker dominance indices higher in the farms than in the natural areas in almost all eco-climatic zones, and by evenness indices always higher in the natural areas than in the farms. This difference of diversity between collection sites in natural areas and in farms was also highlighted by the results of the site classification [45]. We analyzed 70 blood-fed females to identify the origin of the blood-meal (Table 6). A positive amplification for vertebrate blood was obtained for 60 abdomens, among which 35 were identified down to species. Ten blood-meals were identified on cattle (*Bos taurus*), 6 on sheep (*Ovis aries*) or mouflon (*Ovis musimon*), 5 on horse (*Equus caballus*), 9 on RD, 2 on roe deer (*Capreolus capreolus*), 1 on chamois (*Rupicapra rupicapra*), and 2 on bird (*Sylvia* genus). Among the 24 females identified morphologically as *C. obsoletus s.l./C. scoticus*, 16 were *C. obsoletus s.s.*, 2 were *C. scoticus,* and 6 were not identified down to species. Two species were found blood-fed on RD: *C. obsoletus s.s.* and *C. achrayi* (Table 6).

#### 3.2.2. Description of *Culicoides* Species Communities in the Different Environments

**Figure 3:**
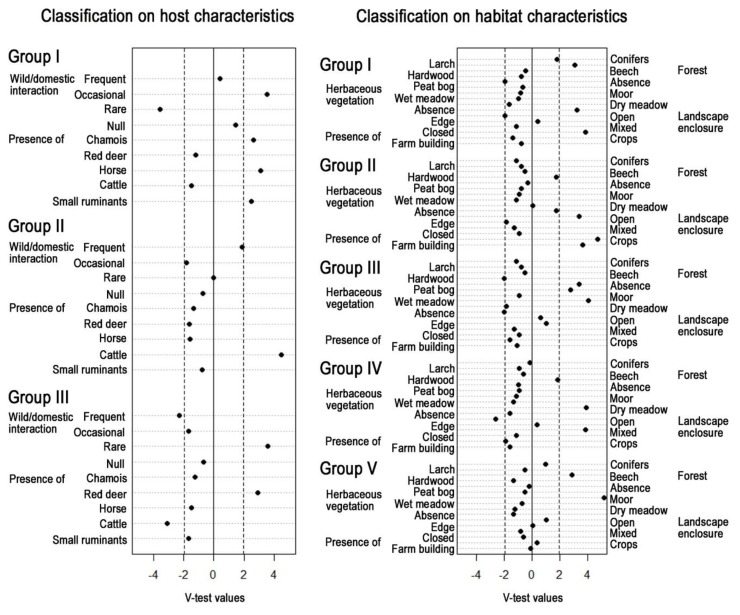
Description of the classifications related to host or environmental characteristics of the collection sites, using test values (measure of the distance between within-group and overall values) for each variable. Groups were obtained using a hierarchical cluster analysis, with the Ward minimum variance method, on the Euclidian distance matrix of the variable coordinates in a multiple correspondence analysis on host or habitat characteristics.

From the 28 collection sites selected, three groups were constituted according to their host characteristics. This partition highlighted a gradient from frequent contacts between cattle and roe deer in both natural and farm environments (group II, *N* = 10)—roe deer were present in all collection sites, to rare contacts between RD and domestic animals (group III, *N* = 9) in natural areas, through an intermediate situation (group I, *N* = 9) characterized by occasional contacts between domestic animals (horse and small ruminants, but not cattle) and wild ruminants (roe deer and chamois, but not RD) (Figure 3). 

Five groups were constituted according to their habitat characteristics (Figure 3). Group II (*N* = 6) was characterized by the presence of crops and farm buildings and by opened landscape. All collection sites located in farms (*N* = 5) belonged to this habitat group. Group I (*N* = 5) was characterized by a close landscape, in larch forest in the absence of herbaceous vegetation; group III (*N* = 6) by the absence of forest and by a vegetation association dominated by wet meadow or peat bogs; group IV (*N* = 8) by an edge or open landscape of dry meadow surrounded by hardwood forest; and group V (*N* = 3) was associated with moors and beech forest.

**Figure 4:**
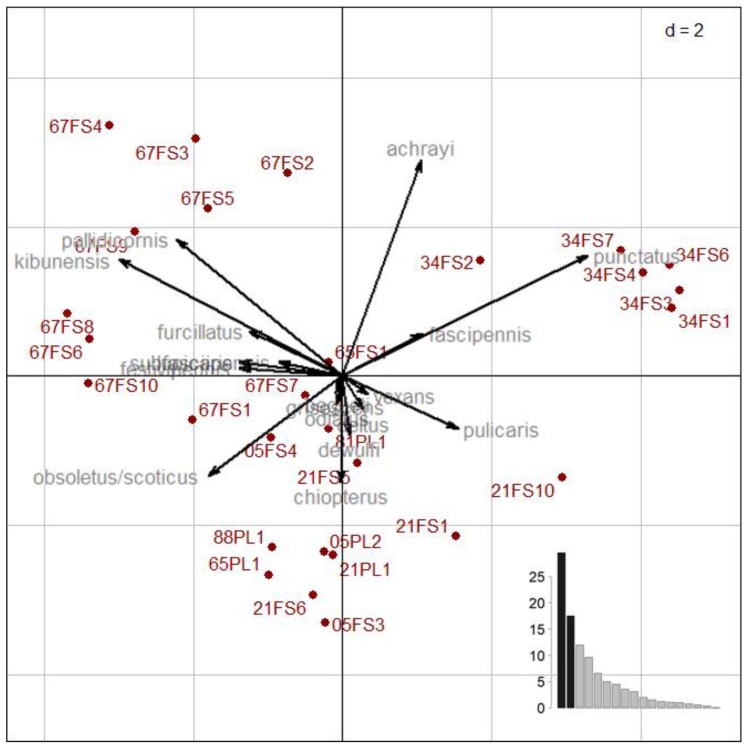
Scatter plot (two first axes) of the principal component analysis (dudi.pca function) performed on the proportion of *Culicoides* per species and site transformed as ordinal classes (encoded 0 as absent, 1 as very rare (0–2%), 2 as rare (2–5%), 3 as secondary (5–20%), 4 as co-dominant (20–60%), and 5 as dominant (>60%)). The drivers of the global species community structure were first the habitat characteristics, which explained 37% of the PCA inertia (*p* = 0.001 using a permutation test), then the eco-climatic zones and the altitude, which were intertwined and explained respectively 35% and 32% of the PCA inertia (*p* = 0.001), respectively, and finally the host characteristics with 13% (*p* = 0.035) and the survey location with 8% (*p* = 0.022).

The cumulative projected inertia of the PCA, carried out on the relative proportions of the 18 most abundant species, was 47% on the two first axes, and 69% on the four first axes (Figure 4). The first axis was structured by the opposition between, on one hand, *C. punctatus* and, on the other hand, *C. pallidicornis*, *C. kibunensis,* and *C. obsoletus s.l./C. scoticus*. The second axis was structured by the opposition between, on one hand, *C. achrayi* and, on the other hand, *C. chiopterus* and secondary *C. obsoletus s.l./C. scoticus* (Figure 4).

**Figure 5:**
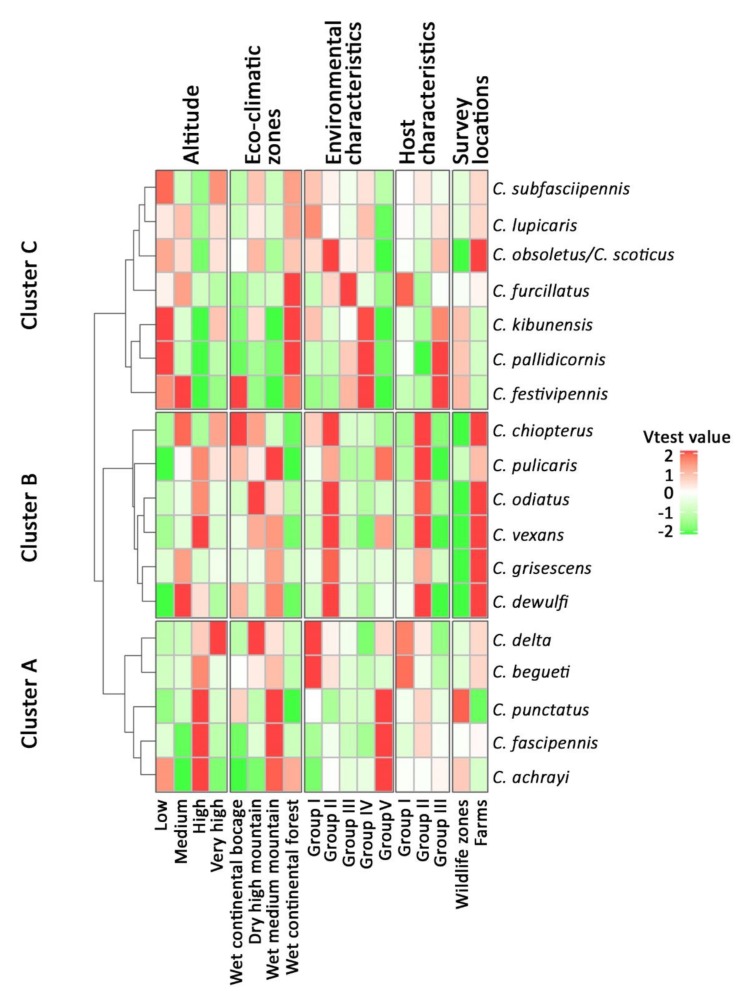
Description of the *Culicoides* species communities by ecological drivers, i.e., eco-climatic zones, altitude, habitat, and host characteristics and survey location, using a heat map of the V-test values (measure of the distance between within-group and overall values). *V*-test values >2 or <−2 (i.e., *p* < 0.05) were rescaled to 2 and −2 to increase the contrast between values located within this interval (i.e., *p* > 0.05).

Crossing the structure of species communities and the ecological factors in collection sites highlighted three clusters of *Culicoides* species (Figure 5). Cluster B was dominant in cattle farms, located in open landscapes with crops and farm buildings. *Culicoides* species of cluster B may be recorded in different eco-climatic zones depending on the species, but not in the wet continental forest; at medium altitude (300–500 m) for *C. chiopterus*, *C. grisescens,* and *C. dewulfi*, and at high altitude (750–1150 m) for *C. odiatus*, *C. vexans,* and *C. pulicaris*. The cluster was found from high altitude (750–1150 m) in wet medium mountains for *C. begueti*, *C. punctatus*, *C. fascipennis*, and *C. achrayi* (which can also be present in wet continental forest) to very high altitude (>1700 m) in dry high mountains for *C. delta*. *Culicoides delta* and *C. begueti* were mainly found in landscapes of larch forest and absence of herbaceous vegetation. *C. punctatus*, *C. fascipennis,* and *C. achrayi* were mainly reported in habitats presenting moors and beech forest. *Culicoides* of cluster C were mostly trapped in the wet continental forest from low to medium altitude (0–500 m), associated with wet meadows for *C. furcillatus* and with edge or open landscapes of dry meadow surrounding by hardwood forest, where RD dominated in absence of interaction with domestic ruminants (especially cattle) for *C. festivipennis*, *C. pallidicornis*, and *C. kibunensis* The presence in this group of the morphologically closely related species *C. obsoletus s.l.*/*C. scoticus* highlighted their ubiquity, as they were dominant in open landscapes dominated by crops linked to farm environments, but could also be extremely abundant in forest environments.

## 4. Discussion

The first issue addressed by this study was the role of RD in the spread of BTV strains. Along this 8-year retrospective study, both PCR examinations and specific VNT (targeting one or another serotype) highlighted that the same serotype infected both RD and domestic ruminants within the same department. In particular, we confirmed that BTV1 did not spread outside the south-western corner of France, where the disease was relatively contained thanks to livestock vaccination (Figure 1, Table 2). The VNT analyses targeting both serotypes revealed the exposure of RD to BTV8 in south-western France (departments 64 and 65, Table 3a), which was not detected by RT-qPCR (Table 2b). Nevertheless, livestock was also exposed to both serotypes in these departments (Table 1, Figure 1). In Corsica, NA targeting BTV1 were only observed by 2013, some seropositive but PCR negative adult animals were observed in 2011 and 2012, which we interpreted as a remainder of the previous BTV strains incursions (i.e., in the early 2000s). These results support the hypothesis that RD populations were secondarily exposed to BTV strains circulating in domestic hosts, and did not have the capacity to spread BTV in natural environments over a long distance (i.e., from one department to another). This conclusion may be surprising, since the distribution of this wildlife species is relatively continuous along forest corridors at the national scale (Figure 2) and has the capacity of long distance dispersal or movements [46]. Some explanations may thus rely rather on the vector and wild host interface than on wildlife behavior (see below).

To be exposed to BTV infection from domestic ruminants, RD populations need to be in contact with *Culicoides* species able to be present in both wildlife areas and farm environments. We used UV-light trap collections to explore the species communities in various environments, from farms to natural areas. Exploring *Culicoides* diversity is difficult due to their large taxonomic diversity and their huge abundance, which may deeply vary from one day to another or from one site to another. Moreover, the UV-light suction trap has a short attraction range and is the most efficient close to animals, leading to difficulties in collecting *Culicoides* in natural environments. Due to these biological and methodological constraints, we decided to follow a descriptive approach to define species communities and to avoid over interpretation of the data. We highlighted that ecological, climatic, and habitat factors were the main drivers of the species community composition, likely through the presence of larval habitats. For instance, *C. chiopterus* and *C. dewulfi* are associated with farms as dung-breeders; *C. vexans* is known to breed in pastures; *C. delta*, *C. fascipennis*, and also *C. grisescens* in peat bogs; and *C. pallidicornis* and also *C. subfasciipennis* in wet meadows [47]. Other collected species, associated with the edges of water bodies (or with tree holes for *C. begueti*), and the *C. obsoletus s.l.*/*C. scoticus* species, which are known to breed in various larval habitats, may thus be found in various environments [47]. Moreover, *Culicoides* species, able to act as bridge vector species between domestic and wild ruminants, need a sufficiently large host-feeding range. Blood-meal analyses confirmed the opportunistic feeding behavior of *C. pulicaris* and *C. kibunensis,* which are able to feed on both mammals and birds, as already known for several primary mammal-feeders such as *C. obsoletus*, *C. punctatus*, or *C. pulicaris* [48], or primary bird-feeders such as *C. festivipennis* or *C. kibunensis* [6,49,50]. We confirmed that species such as *C. obsoletus s.s.* and *C. achrayi* were able to feed on both domestic and wild ruminants, including sheep (and/or mouflons), cattle, roe deer, or RD and then, at least for *C. obsoletus s.s.*, a probable BTV vector species [51] dominant in farms [33], were able to act as bridge vector species. The exposure of RD populations to BTV infection from domestic ruminants depends on the dispersal ability of *Culicoides* species from farm to forest environments and on the presence of RD in open areas, which are both highly dependent on the landscape structure. These results are consistent with the first observations from Rossi et al. [13], who reported the important exposure of RD populations to BTV8 in 2008 in landscapes exhibiting a patchy forest pattern with an important overlap with pastures or other open lands and a low BTV8 exposure of RD populations in forests with a high degree of canopy closure.

The second question of BTV8 maintenance in wild hosts is a more complex issue to address. The BTV maintenance cycle should involve a sufficiently high density of competent vector species. Several species, such as *C. furcillatus*, *C. achrayi*, *C. pallidicornis* or *C. kibunensis*, are associated with forest environments inhabited by RD populations. The absence of information of their vector competence limits our ability to conclude that these species could act as enzootic vectors within RD populations. On the contrary, we confirmed that *C. obsoletus s.s.*, a probable BTV vector species, may feed on RD and may be extremely abundant in forest environments. One should thus expect an efficient BTV sylvatic cycle. However, the efficiency of transmission may depend on the intensity of permanent contacts between RD and *Culicoides* populations, which could be limited by RD heterogeneous spatial repartition and/or density.

The interest of the present study was the longitudinal monitoring of RD populations combining PCR and serological examinations. PCR positive results were observed zero to 1 year after the apparent disease fade out in livestock (in 2008 or 2009, according to the continental department). RNA positive results (virus isolation negative/inconclusive) do not demonstrate that RD were still infectious at the time of sampling. Indeed, BTV viremia is not well known in that species [14], but is possibly similar to the viremia observed in the domestic cattle (i.e., up to 4 weeks after infection, [52]). The duration of RNAemia in RD appears to be long (greater than 100 days) [14] and similar to those observed in domestic ruminants (up to 157 days in cattle) [52]. We may thus consider that most of the PCR positive results observed in 2009 corresponded to virus circulation during the current vector season (even though we cannot rule out that some of them got infected by 2008). After 2009, no PCR positive results were observed in the continent, which could have arisen either because of BTV fade out in RD or a lack of power of our data based on small sample sizes. The investigation of antibody dynamics and NA titers were therefore useful to complete the analysis.

At first sight, the presence of antibodies in young RD (aged from 6 to 18 months), observed 2 to 3 years after the last domestic outbreak in some departments, could suggest a recent BTV circulation and a proper capacity of that species to maintain a sylvatic cycle for a couple of years. However, we also investigated the alternative scenario of maternal antibodies portage later than 6 months in some of the young RD. This hypothesis seemed reasonable, since the initial antibody titers rose to relatively high values (Table 3c), which may have generated long-lasting maternally-derived antibodies in the offspring of some aged does. Furthermore, in spite of contrasting initial exposure among the sampled RD populations, some being up to 60% in 2008 (Table 2), seroprevalence and NA titers continuously decreased over time among the sampled RD populations (Table 2 and Table 3c). A different temporal trend of seroprevalence according to the age class was also observed. As previously discussed by Rossi et al. [13,25], the lower seroprevalence observed initially in young compared to adult RD may reflect a lower initial exposure of young individuals due to a shorter period of exposure, the hider behavior of fawn, and/or a lower attractiveness of small animals regarding vectors compared to bigger/older ones. However, none of these factors explain why seroprevalence decreased much faster in the young and subadult RD (i.e., less than 2 years old) compared to adults, resulting in no more seropositive results in these population groups after 2012. Furthermore, no positive PCR results were reported among these seropositive RD of less than 2 years old after 2009. The analysis of NA titers provided additional information: Not only did NA titers decrease over time whatever the zone, but also NA titers were, on average, twice as low in the young and subadult RD compared to adults sampled during the same year. These results rather suggest a progressive loss of antibodies in adults and the sporadic presence of long lasting maternal antibodies than a recent exposure of young RD to BTV. In the latter case, this should have resulted in an NA titer increase in these supposedly recently infected animals. We thus conclude that the young seropositive we observed after 2009 in the continental departments were likely to carry maternal antibodies and that BTV did not circulate more than 1 year after the last domestic outbreak within the same department. This conclusion is interesting to confront with the classic statement that young seropositive reflect the recent disease circulation in wildlife populations, a hypothesis that resulted invalidated in the present study and in some others (see Saubusse et al. [53] for a comparable situation in wild boar). Surprisingly, lower NA titers in males were observed compared to females, while males were initially much more exposed than females to BTV1 or 8 [13]. This apparent contradiction possibly arose because male RD survive less than females within harvested populations [54], resulting in adult bucks being less aged than adult does and thus having lower NA titers. Overall, these results support the hypothesis of no recent exposure in RD populations and the incapacity of RD populations and *Culicoides* associated populations to maintain a sylvatic cycle on their own. 

Our third question concerned the possible spillback of BTV8 from RD to domestic ruminants as an explanation of BTV8 re-emergence in August 2015 (Zone A). The absence of long-term BTV circulation within the 12 sampled continental RD populations invalidated this hypothesis. Furthermore, no positive PCR and no increase in VNT were observed in the three RD populations inhabiting Zone A (i.e., where BTV8 re-emerged in 2015–2016 in livestock), suggesting that the virus had not yet spread to wildlife by the autumn 2015. This lack of circulation in wild populations possibly arose due to the low incidence observed in the domestic animals at that time (Table 2) [55]. Indeed, in departments where BTV circulation was evidenced in 2015–2016, only 3% of young cattle (used as an indicator of the level of recent viral circulation) were seropositive [55], while seroprevalence from 4% up to 100% were observed in 2006–2008 [56]. The re-emergence of BTV8 in 2015 in Central France was thus more likely to have been caused by the low circulation of BTV8 in cattle between 2010 and 2015, such as suggested by the retrospective study of Courtejoie et al. [19].

The factors influencing the role of RD as a maintenance or spill over host at the European level is obviously a different issue. In Spain, studies reported the occurrence of outbreaks in RD in areas with no domestic cases [22] and long-term presence of both ARNemia and antibodies in young RD, thus advocating the potential maintenance role of that wild species [9,23,24]. The situation observed in Central–Southern Spain is possibly different from the French one due to the presence of abundant populations of *C. imicola* [6,24]. Indeed, we cannot exclude that *C. imicola* populations in Spain exhibit higher vector competence for BTV than the Palearctic *Culicoides* species. Furthermore, persistence could also rely on the very high RD density reported in these region (i.e., up to 20 RD/km^2^) [24], constituting a particular niche at the European level [9]. 

In the present study, the populations sampled in some hunting estates exhibited high initial exposure and relatively high densities (5–10 RD/km^2^). However, even in these estates, we observed no BTV RNA persistence for longer than a year after the completion of livestock vaccination. Some populations were sampled in Mediterranean environments (Alpes-Maritimes department and Corsica Island) but did not exhibit long-term BTV persistence, even if, for some locations in southern Corsica, *C. imicola* populations may reach the same level of abundance as in southern Spain. These results suggest that for a combination of eco-environmental factors (e.g., open landscape, high temperatures), vectors community and high RD density are certainly necessary for BTV to persist in natural ecosystems.

## 5. Conclusions

This study highlights the interest of long-term wildlife surveillance and the appropriate (professional) storage of sera/organ banks at a national level, which requires a sound collaboration between national agencies, laboratories, researchers, biologists, farmers, and hunting organizations. We confirmed that BTV strain distributions were alike between wild and domestic populations, and that RD played no role in spreading BTV strains between departments. The absence of RNA detection more than 1 year after the last domestic outbreak, and the decrease of seroprevalence and NA titers in all sampled RD populations over time, suggest that no persistent sylvatic cycle could settle after the fade out of BTV1 or BTV8 in livestock. In some areas, positive serological results occurred in young RD up to 3 years after the last observed domestic outbreak, but their low NA titers compared to adults sampled the same year suggest that they were more likely to carry maternal antibodies than to have been recently infected. We also did not find evidence that RD populations had been recently infected in the area where BTV8 had re-emerged in livestock by 2015, which certainly rather relied on a low-level circulation of BTV8 in cattle from 2010 up to 2015. We conclude that BTV outbreaks have been self-limiting in free-ranging RD (continent and Corsica) that did not play the role of maintenance host in spite of a high initial exposure and relatively high RD densities in some areas. The study of *Culicoides* community composition confirmed previous studies from Spain and the Czech Republic stating that, despite different species communities in natural and farm environments, some ubiquitous species may be abundant to both environments and act as bridge vector species. The absence of BTV persistence in RD populations may thus be more likely due to ecological factors limiting the interactions between RD and *Culicoides* populations rather than to the absence of competent vector species in forest environments. A retrospective analysis including more countries, exploring NA titer to disentangle maternal antibody carriers from recently exposed individuals, and describing *Culicoides* communities in various natural environments, could help to better assess the role of RD in BTV maintenance at the European level.

## Figures and Tables

**Table 1:** Number of domestic Bluetongue virus (BTV) outbreaks occurring in domestic farms within the 15 departments considered in the study. “Dep” corresponds to the department number (located in Figure 1). No BTV outbreak was observed from 2010 to 2013. Zone A corresponds to BTV8 re-emergence in livestock in 2015, while Zone B corresponds to the continental departments with no apparent re-emergence. * departments with BTV8 outbreaks in domestic ruminants in 2015.

Department	Zone	2008–2009	2009–2010	2010–2013	2013–2014	2014–2015	2015–2016
05 Hautes-Alpes	B	6 (BTV8)	0	0	0	0	0
06 Alpes Maritimes	B	3 (BTV8)	0	0	0	0	0
21 Côte d’Or	B	593 (BTV8)	0	0	0	0	0
2A Corse du Sud	/	0	0	0	80 (BTV1)	0	0
2B Haute-Corse	/	0	0	0	89 (BTV1)	0	0
36* Indre	A	560 (BTV8)	0	0	0	0	1 (BTV8)
41 Loir-et-Cher	B	177 (BTV8)	0	0	0	0	0
45* Loiret	A	102 (BTV8)	0	0	0	0	1 (BTV8)
52 Haute-Marne	B	313 (BTV8)	1 (BTV8)	0	0	0	0
57 Moselle	B	116 (BTV8)	0	0	0	0	0
58* Nièvre	A	1169 (BTV8)	2 (BTV8)	0	0	0	7 (BTV8)
60 Oise	B	28 (BTV8)	0	0	0	0	0
64 Pyrénées Altantiques	B	412 (BTV1, 8)	0	0	0	0	0
65 Hautes Pyrénées	B	115 (BTV1, 8)	0	0	0	0	0

**Table:** **(a) ELISA results observed among 3065 RD sera.**

Year		2008–2009		2009–2010		2010–2011		2011–2012		2012–2013		2013–2014		2014–2015		2015–2016	
Zone	Dep	pos	tot	pos	tot	pos	tot	pos	tot	pos	tot	pos	tot	pos	tot	pos	tot
B	05	1	11	0	45												
B	06							0	1	1	13						
B	21	32*	46	23*	49			10*	34	5	13						
/	2A									4	15	5*	12				
/	2B							2	23	0	33	20*	33			11	65
A	36					59*	122	34*	117					3	55		
B	41	40*	62	27*	108	12*	54	20*	130	17	101	16	93	15	115	11	50
A	45					7	14	1	11	1	9	0	6				
B	52	32*	47	16*	39			4*	40	9*	63						
B	57	5*	33	6	32	1	21	1	25								
A	58									3	28	1	40	2	27	0	12
B	60							7	68	3	71						
B	64	14*	23	4	20			4	30	3	21						
B	65	7	19	19*	52	4	17	12	49	5	33						
B	67	5	39	7	62	1	41	2	56	1	62						

**Table:** **(b) PCR results observed among 458 c-ELISA positive RD (spleens or full blood).**

Zone	Dep	Result	Type	2008–2009	2009–2010	2010–2011	2011–2012	2012–2013	2013–2014	2014–2015	2015–2016
/	2A	neg	BTV1	-	-	-	-	4	9	-	-
		pos		-	-	-	-	**0**	**3**	-	-
/	2B	neg	BTV1	-	-	-	20	-	22	-	5
		pos		-	-	-	**0**	**-**	**11**	-	**0**
B	64	neg	BTV1	0	4	-	4	3	-	-	-
		pos		**14**	**0**	-	**0**	**0**	-	-	-
B	65	neg	BTV1	0	18	4	6	5	-	-	-
		pos		**8**	**1**	**0**	**0**	**0**	-	-	-
B	21	neg	BTV8	0	26	-	9	5	-	-	-
		pos		**32**	**7**	-	**0**	**0**	-	-	-
B	41	neg	BTV8	4	2	12	20	17	16	-	5
		pos		**36**	**25**	**0**	**0**	**0**	**0**	-	**0**
B	52	neg	BTV8	9	15	-	4	9	-	-	-
		pos		**23**	**1**	-	**0**	**0**	-	-	-
B	57	neg	BTV8	0	3	1	1	-	-	-	-
		pos		**5**	**3**	**0**	**0**	-	-	-	-
B	60	neg	BTV8		-	-	7	4	-	-	-
B	67	neg	BTV8	0	5	1	2	1	-	-	-
		pos		**5**	**2**	**0**	**0**	**0**	-	-	-
Total of RD PCR tested % of BTV8 PCR positive				136	112	18	73	48	61	0	10
				**90.4**	**34.8**	0	0	0	**23**	0	0

**Table:** **(a) VNT titers in 331 sera tested regarding BTV8.**

VNT Titers		0	4	8	16	32	64	128	256
zone	dep								
B	05					1	3		
B	06			1					
B	21	2	3	3	4	10	8	3	1
/	2B*	2*							
A	36	7	7	32	51	15	8	5	
B	41	5	9	19	25	17	16	3	
A	45			2	3	1	2	1	
B	52		2	2	2	6	3	1	
B	57					1	3		
A	58		1	3		3			
B	60			1	3	5	1		
B	64	1	2		4	3			
B	65					1	3		
B	67				4	3	1	3	

* Department without BTV8 case reported in livestock.

**Table:** **(b) VNT titers in 103 sera c-ELISA positive tested regarding BTV1.**

VNT Titers		0	16	32	64	128	256
zone	dep						
B	21*	6*					
/	2A	9	2		1		
/	2B	13	7	7	4	2	1
A	36*	38*					
A	41*	5*					
B	52*	3*					
B	64	1	2	1	1		

* Department without BTV1 case reported in livestock.

**Table:** **(c) Decrease over time of VNT titers regarding BTV8 in 331 c-ELISA positive sera.**

Year/VNT Titers	0	4	8	16	32	64	128	256
2008–2009				14	23	23	9	1
2010–2011	2	3	17	30	10	5	4	
2011–2012	10	9	20	31	18	11	3	
2012–2013	1	3	10	8	11	3		
2013–2014		4	3	5		5		
2014–2015		2	7	6	4	1		
2015–2016	4	3	6	2				

**Table 4:** No. *Culicoides* collected in natural areas (June to August 2008) and in farms (2009–2012) using UV-light suction traps (OVI model) in five different ecological and climatic French regions.

Species	Collections in Natural Zones							Collections in Farms							All Locations		
	Alsace Region (10 Sites, 11 Collections)	Bourgogne Region (10 Sites, 11 Collections)	Alps Region (5 Sites, 6 Collections)	Haut-Languedoc Region (8 Sites, 26 Collections)	Pyrenees Region (1 site, 1 Collection)	Total	Rank	Alsace Region (1 site, 85 Collections)	Bourgogne Region (1 site, 92 Collections)	Alps Region (1 site, 50 Collections)	Haut-Languedoc Region (1 site, 100 Collections)	Pyrenees Region (1 site, 95 Collections)	Total	Rank	Weighted Average Collection^b^	Rank	Cumulative Percentage
*C. obsoletus/C. scoticus*	29,774^a^	435	34	75	71	**30,389**	**1**	3990	634	3661	2275	844	**11,403**	**1**	**1587.2**	**1**	**70.55%**
*C. furcillatus*	9087	0	1	4	2	**9094**	**2**	50	3	66	51	7	**176**	**8**	**151.4**	**2**	**77.28%**
*C. achrayi*	2787	1	0	1226	6	**4020**	**3**	20	34	10	376	0	**441**	**3**	**103.2**	**3**	**81.86%**
*C. chiopterus*	20	8	1	0	0	**29**	**19**	98	600	11	20	6	**735**	**2**	**73.9**	**4**	**90.40%**
*C. punctatus*	269	99	1	2692	83	**3144**	**4**	32	10	32	24	5	**102**	**11**	**56.5**	**5**	**84.37%**
*C. pulicaris*	112	52	1	56	0	**221**	**9**	175	5	50	131	31	**391**	**4**	**42.4**	**6**	**93.83%**
*C. lupicaris*	363	3	2	10	40	**418**	**8**	202	2	18	23	44	**287**	**5**	**34.8**	**7**	**91.95%**
*C. pallidicornis*	1653	0	0	0	4	**1657**	**5**	15	0	0	19	8	**42**	**15**	**28.6**	**8**	**85.65%**
*C. dewulfi*	14	34	0	5	4	**57**	**16**	71	44	1	5	95	**216**	**6**	**22.4**	**9**	**94.83%**
*C. vexans*	0	0	0	51	0	**51**	**17**	2	2	204	3	0	**211**	**7**	**21.8**	**10**	**95.80%**
*C. kibunensis*	720	6	12	6	10	**754**	**6**	35	4	24	15	2	**78**	**12**	**18.9**	**11**	**86.49%**
*C. odiatus*	0	0	0	0	0	**0**		0	0	151	12	4	**167**	**9**	**16.7**	**12**	**97.30%**
*C. grisescens*	0	0	0	0	0	**0**		149	0	0	0	0	**149**	**10**	**14.9**	**13**	**97.96%**
*C. festivipennis*	481	264	0	3	2	**750**	**7**	6	14	4	3	5	**31**	**18**	**14.1**	**14**	**87.11%**
*C. delta*	24	0	10	82	5	**121**	**12**	2	1	3	44	0	**50**	**13**	**6.8**	**15**	**96.34%**
*C. fascipennis*	53	0	0	102	0	**155**	**10**	0	0	3	28	1	**32**	**16**	**5.5**	**16**	**96.04%**
*C. subfasciipennis*	137	0	1	1	0	**139**	**11**	7	1	0	14	7	**29**	**21**	**4.9**	**17**	**96.56%**
*C. poperinghensis*	0	0	0	0	0	**0**		1	2	15	19	11	**48**	**14**	**4.8**	**18**	**98.67%**
*C. newsteadi*	0	17	0	0	0	**17**	**20**	2	6	16	4	3	**31**	**18**	**3.3**	**19**	**98.46%**
*C. flavipulicaris*	0	0	0	0	0	**0**		0	0	32	0	0	**32**	**16**	**3.2**	**20**	**99.04%**
*C. nubeculosus*	0	0	0	0	0	**0**		12	0	15	2	2	**31**	**18**	**3.1**	**21**	**99.17%**
*C. brunnicans*	0	0	0	8	0	**8**	**25**	1	8	2	7	8	**26**	**22**	**2.7**	**22**	**98.89%**
*C. fagineus*	38	0	0	0	0	**38**	**18**	0	0	0	20	0	**20**	**25**	**2.6**	**23**	**98.31%**
*C. circumscriptus*	0	0	0	1	0	**1**	**29**	0	0	16	8	1	**25**	**23**	**2.5**	**24**	**99.28%**
*C. picturatus*	12	0	0	1	0	**13**	**21**	1	2	13	4	2	**22**	**24**	**2.3**	**25**	**98.78%**
*C. parroti*	0	0	0	0	0	**0**		9	4	0	0	7	**20**	**25**	**2.0**	**26**	**99.43%**
*C. begueti*	0	0	0	0	105	**105**	**13**	0	3	1	0	0	**4**	**38**	**1.9**	**27**	**98.05%**
*C. sejfadinei/C. tauricus*	0	0	0	0	0	**0**		0	0	19	0	0	**19**	**27**	**1.9**	**28**	**99.52%**
*C. impunctatus*	9	0	0	61	0	**70**	**15**	2	0	0	6	0	**8**	**34**	**1.8**	**29**	**98.20%**
*C. clastrieri*	84	0	0	0	0	**84**	**14**	0	2	0	1	1	**4**	**38**	**1.6**	**30**	**98.12%**
*C. simulator*	0	0	0	0	0	**0**		0	12	3	0	0	**15**	**28**	**1.5**	**31**	**99.65%**
*C. alazanicus*	0	1	0	0	0	**1**	**29**	0	1	0	12	1	**14**	**29**	**1.4**	**32**	**99.71%**
*C. segnis*	1	0	0	3	0	**4**	**27**	2	1	9	2	0	**14**	**29**	**1.4**	**33**	**99.58%**
*C. cataneii/C. gejgelensis*	0	11	0	0	0	**11**	**22**	0	0	3	8	1	**12**	**31**	**1.4**	**34**	**99.35%**
*C. longipennis*	0	0	0	0	0	**0**		0	0	11	0	0	**11**	**32**	**1.1**	**35**	**99.76%**
*C. tauricus*	0	0	0	0	0	**0**		11	0	0	0	0	**11**	**32**	**1.1**	**35**	**99.81%**
*C. subfagineus*	0	0	0	0	0	**0**		0	0	5	1	0	**6**	**35**	**0.6**	**37**	**99.84%**
*C. pictipennis*	0	0	0	0	0	**0**		3	1	0	0	2	**6**	**35**	**0.6**	**38**	**99.87%**
*C. maritimus*	0	0	0	0	0	**0**		0	0	0	5	0	**5**	**37**	**0.5**	**39**	**99.89%**
*C. duddingstoni*	0	0	0	0	0	**0**		0	4	0	0	0	**4**	**38**	**0.4**	**40**	**99.91%**
*C. stigma*	1	0	0	0	0	**1**	**29**	2	1	0	0	0	**3**	**41**	**0.3**	**41**	**99.92%**
*C. minutissimus*	0	0	0	0	0	**0**		0	1	1	0	1	**3**	**41**	**0.3**	**42**	**99.95%**
*C. riethi*	0	0	0	0	0	**0**		3	0	0	0	0	**3**	**41**	**0.3**	**43**	**99.96%**
*C. dendriticus*	0	0	0	2	0	**2**	**28**	0	0	0	2	0	**2**	**44**	**0.2**	**44**	**99.93%**
*C. puncticollis*	0	0	0	0	0	**0**		0	0	2	0	0	**2**	**44**	**0.2**	**45**	**99.97%**
*C. salinarius*	0	0	0	0	0	**0**		2	0	0	0	0	**2**	**44**	**0.2**	**45**	**99.98%**
*C. santonicus*	0	0	0	0	0	**0**		0	0	0	0	2	**2**	**44**	**0.2**	**47**	**99.98%**
*C. clintoni*	10	0	0	0	0	**10**	**23**	0	0	0	0	0	**0**		**0.1**	**48**	**99.81%**
*C. cameroni*	0	0	7	0	0	**7**	**26**	0	0	0	0	0	**0**		**0.1**	**49**	**99.82%**
*C. jumineri*	0	0	0	0	0	**0**		0	0	0	1	0	**1**	**48**	**0.1**	**50**	**99.99%**
*C. gornostaevae*	0	0	0	0	0	**0**		1	0	0	0	0	**1**	**48**	**0.1**	**50**	**99.99%**
*Culicoides* sp.	0	0	7	2	0	**9**		0	0	0	0	1	**1**		**0.2**		**100.00%**
**Total**	**45,649**	**931**	**77**	**4391**	**332**	**51,380**		**4903**	**1399**	**4399**	**3141**	**1099**	**14,942**		**2250**		

^a^ Total numbers are presented after having been aggregated against time, using the maximum number of *Culicoides* recorded per species and per collection site for natural areas, and using the yearly mean of the maximum number of *Culicoides* recorded annually per species and per collection site. ^b^ This corresponds to the average of a mean collection in natural areas (34 sites) and a mean collection in farms (5 sites).

**Table 5:** Description of the *Culicoides* species communities (observed and extrapolated species richness, alpha of log series, the Berger–Parker dominance, and the evenness indices) collected in natural zones (June to August 2008) and in farms (2009–2012) using UV-light suction traps (OVI model) in five different ecological and climatic French regions.

	Collections in Natural Areas				Collections in Farms			
	WCF^a^	WCB^a^	DHM^a^	WMM^a^	WCF^a^	WCB^a^	DHM^a^	WMM^a^
No. collections (sites x trapping)	12	10	5	18	50	67	25	136
Observed no. species	21	12	10	21	29	27	30	36
Chao expected no. species ± s.e.^b^	24.67 ± 4.88	12.60 ± 1.20	19.80 ± 10.62	26.90 ± 7.16	33.08 ± 4.79	39.31 ±10.53	57.00 ± 20.09	41.03 ± 4.41
Fisher α of log series	2.103	1.944	3.193	2.830	4.091	4.761	4.343	5.441
Berger-Parker dominance index	0.652	0.467	0.486	0.588	0.814	0.444	0.830	0.724
Evenness index	0.377	0.590	0.680	0.425	0.271	0.387	0.258	0.357

^a^ WCF, wet continental forest; WCB, wet continental bocage; DHM, dry high mountain; WMM, wet medium mountain. ^b^ s.e., standard error.

**Table 6:** Identification of the blood-meal origin of the blood-fed females collected in wildlife zones (June to August 2008) using UV-light suction traps (OVI model) in five different ecological and climatic French regions.

Species	No. Blood-Fed Females	No. Positive for Vertebrate Blood	No. Females Found Fed on										
			Red Deer *Cervus elafus*	Roe Deer *Apreolus capreolus*	Chamois *Rupicapra rupicapra*	Cattle *Bos taurus*	Goat *Capra hircus*	Sheep or Mouflon *Ovis aries*	Horse *Equus caballus*	Pig or Wild Boar *Sus scrofa*	Cat *Felis catus*	Dog *Canis lupus familaris*	Birds *Sylvia* genus
*C. achrayi*	15	14	6	1		2		1					
*C. dewulfi*	2	2				2							
*C. fascipennis*	1	1											
*C. festivipennis*	2	2											
*C. kibunensis*	2	2			1								1
*C. lupicaris*	4	3											
*C. obsoletus/C. scoticus*	6	5	1			1			1				
*C. obsoletus*	16	13	2	1		2		1					
*C. scoticus*	2	2				1							
*C. pulicaris*	1	1											1
*C. punctatus*	10	7				1		1	3				
*C. segnis*	1	1				1							
*C. vexans*	8	7						3	1				
**Total**	**70**	**60**	**9**	**2**	**1**	**10**	**0**	**6**	**5**	**0**	**0**	**0**	**2**

## Appendix A

Table A1 and Table A2 describes the complete models and the QAICc from the best models: A1 for seroprevalence and A2 for VNT. The dredge function allowed us to test all possible nested (simpler) models, including the simple effects and also first order interactions; the most complete model tested for each variable is explicated in the first line of Table A1 and Table A2. Please notice that for summarizing the most interesting information, we only described the best models (i.e., lowest QAICc in a delta of two) within the two tables, and we finally retained the model with less co-variables, according to the principle of parsimony.

**Table A1:** QAICc of the two best models retained by the dredge function regarding c-LISA seroprevalence (i.e., lowest QAICc in a delta of two). The more parsimonious model is model 1 (*in italics*) and was finally retained.

Model	Age	Dep	Season	Sex	Age*season	Dep*season	df	logLik	QAICc	Delta
*1*	*+*	*+*	*−0.373*	*NA*	*+*	*NA*	*18*	*−799.397*	*1540.870*	*0*
2	+	+	*−*0.373	+	+	NA	19	*−*799.397	1542.907	2.037

**Table A2:** QAICc of the four best models tested using the dredge function regarding VNT (i.e., lowest QAICc in a delta of two). The more parsimonious model is model 1 (*in italics*) that was finally retained.

Model	Age	Dep	Season	Sex	Age*dep	Age*season	Dep*season	df	logLik	QAICc	Delta
*1*	*NA*	*NA*	*−0.243*	*NA*	*NA*	*NA*	*NA*	*3*	*−390.775*	*267.471*	*0*
2	NA	NA	*−*0.244	+	NA	NA	NA	4	*−*388.289	267.895	0.424
3	+	NA	*−*0.262	NA	NA	NA	NA	5	*−*386.921	269.077	1.605
4	+	NA	*−*0.197	NA	NA	+	NA	7	*−*382.236	270.193	2.722
